# Using HIV Risk Self-Assessment Tools to Increase HIV Testing in Men Who Have Sex With Men in Beijing, China: App-Based Randomized Controlled Trial

**DOI:** 10.2196/45262

**Published:** 2023-09-01

**Authors:** Qianqian Luo, Zunyou Wu, Guodong Mi, Jie Xu, Sarah Robbins Scott

**Affiliations:** 1 School of Nursing Binzhou Medical University Yantai China; 2 The National Center for AIDS/STD Control and Prevention Chinese Center for Disease Control and Prevention Beijing China; 3 Blued City Holdings, Ltd Beijing China

**Keywords:** app, China, HIV testing, men who have sex with men, risk assessment

## Abstract

**Background:**

Men who have sex with men (MSM) in China hold a low-risk perception of acquiring HIV. This has resulted in an inadequate HIV testing rate.

**Objective:**

This study aims to investigate whether administering HIV risk self-assessments with tailored feedback on a gay geosocial networking (GSN) app could improve HIV testing rates and reduce sexual risk behaviors in Chinese MSM.

**Methods:**

We recruited MSM from Beijing, China, who used the GSN platform Blued in October 2017 in this 12-month double-blinded randomized controlled trial. From October 2017 to September 2018, eligible participants were randomly assigned to use a self-reported HIV risk assessment tool that provided tailored feedback according to transmission risk (group 1), access to the same HIV risk assessment without feedback (group 2), or government-recommended HIV education materials (control). All interventions were remotely delivered through the mobile phone–based app Blued, and participants were followed up at 1, 3, 6, and 12 months from baseline. The number of HIV tests over the 12-month study was the primary outcome and was assessed using an intention-to-treat analysis with an incident rate ratio (IRR). Unprotected anal intercourse (UAI) over 6 months was assessed by a modified intention-to-treat analysis and was the secondary outcome. All statistical analyses were conducted in SAS 9.3 (SAS Institute, Inc.), and a *P* value <.05 was considered statistically significant.

**Results:**

In total, 9280 MSM were recruited from baseline and were randomly assigned to group 1 (n=3028), group 2 (n=3065), or controls (n=3187). After follow-up, 1034 (34.1%), 993 (32.4%), and 1103 (34.6%) remained in each group, respectively. Over 12 months, group 1 took 391 tests (mean of 2.51 tests per person), group 2 took 352 tests (mean of 2.01 tests per person), and controls took 295 tests (mean of 1.72 tests per person). Group 1 had significantly more HIV testing than the control group (IRR 1.32, 95% CI 1.09-4.58; *P*=.01), while group 2 did not differ significantly from the controls (IRR 1.06, 95% CI 0.86-1.30; *P*=.60). The proportion of UAI was not statistically different among different groups, but all 3 groups had UAI, which declined from baseline.

**Conclusions:**

Repeated HIV risk assessments coupled with tailored feedback through GSN apps improved HIV testing. Such interventions should be considered a simple way of improving HIV testing among MSM in China and increasing awareness of HIV status.

**Trial Registration:**

ClinicalTrials.gov NCT03320239; https://clinicaltrials.gov/study/NCT03320239

## Introduction

Consistent with global trends, China has experienced an increase in HIV prevalence among men who have sex with men (MSM), with the national HIV prevalence estimated to be 5.7% across 2001-2018 [1,2]. According to the Chinese Center for Disease Control and Prevention (CDC), MSM accounted for more than 25% of new HIV infections [3] and were responsible for 78% of newly diagnosed infections within Beijing in 2017 [4]. Concerningly, it is estimated that approximately 25% of MSM with HIV do not know their HIV-positive status [5,6]. This is far from the Joint United Nations Program on HIV/AIDS (UNAIDS) target that 90% of people living with HIV know their HIV status [7], creating the need for interventions that link MSM in China to HIV care.

Despite the high burden of HIV infections within MSM communities, HIV testing rates among MSM are low in China, and only half of MSM reported to have ever tested for HIV [8-10]. This is thought to be due to social and psychological barriers, including a lack of trust in facility-based services, hesitancy to use facilities, and anxiety about knowing one’s HIV status [11]. Low perceived risk of HIV transmission is also problematic for HIV testing uptake [12-14], with almost 70% of MSM underestimating their risk of HIV [15]. Underestimation of HIV risk can significantly impact HIV testing, leading to underuse of preventative services and riskier sexual behaviors [16]. This is thought to be exacerbated by the COVID-19 pandemic, where city lockdowns and stay-at-home measures further limited HIV testing service usage [17,18]. Therefore, effective interventions to expand HIV testing in MSM populations and prevent these deterrents are needed.

To improve HIV risk perception among MSM, HIV risk assessment tools have been developed in many countries to motivate HIV testing and the initiation of pre-exposure prophylaxis (PrEP) [19-21]. Remote delivery of HIV-related knowledge, booking services, and monitoring of sexual risk behaviors on gay geosocial networking (GSN) apps have become novel, cost-effective, population-based HIV prevention strategies [22,23], especially in the context of the COVID-19 pandemic. The combination of risk assessment and feedback delivered through GSN could provide a convenient and private way for MSM to become aware of their risk and increase awareness of the need for HIV testing. Therefore, a randomized controlled trial (RCT) was conducted where HIV risk assessment and feedback were delivered by a popular GSN, Blued, in Beijing, China. The goal of this study was to determine the effect of this intervention on HIV testing numbers and unprotected anal intercourse (UAI) among Chinese MSM.

## Methods

### Study Design and Participants

This double-blinded, triple-arm RCT was conducted among MSM in Beijing, China, between October 2017 and September 2018. Participants were recruited in October 2017 through the popular Chinese gay dating app Blued, which has approximately 480,000 monthly active users in Beijing [24]. The inclusion criteria were as follows: MSM who were 18 years or older; had anal intercourse with a man in the past 6 months; self-reported negative or unknown HIV status; resided in Beijing; logged onto the app at least once in the past week before enrollment; and agreed not to share the study materials with others in the study. Participants were excluded if they self-reported injection drug use within 6 months before enrollment, participated in another web-based intervention during the study period, or planned to move away from Beijing within the following year. The trial (ClinicalTrials.gov NCT03320239) was conducted following the CONSORT (Consolidated Standards of Reporting Trials) for reporting clinical trials [25,26].

### Randomization and Masking

Participants were simply randomized in a 1:1:1 ratio by a computerized randomization algorithm with SAS 9.3 (SAS Institute, Inc.) into 3 groups (group 1, group 2, and the control group). The assignment of group allocations was masked to both the study staff and participants to ensure double-blinding.

### Procedures

**Figure 1:**
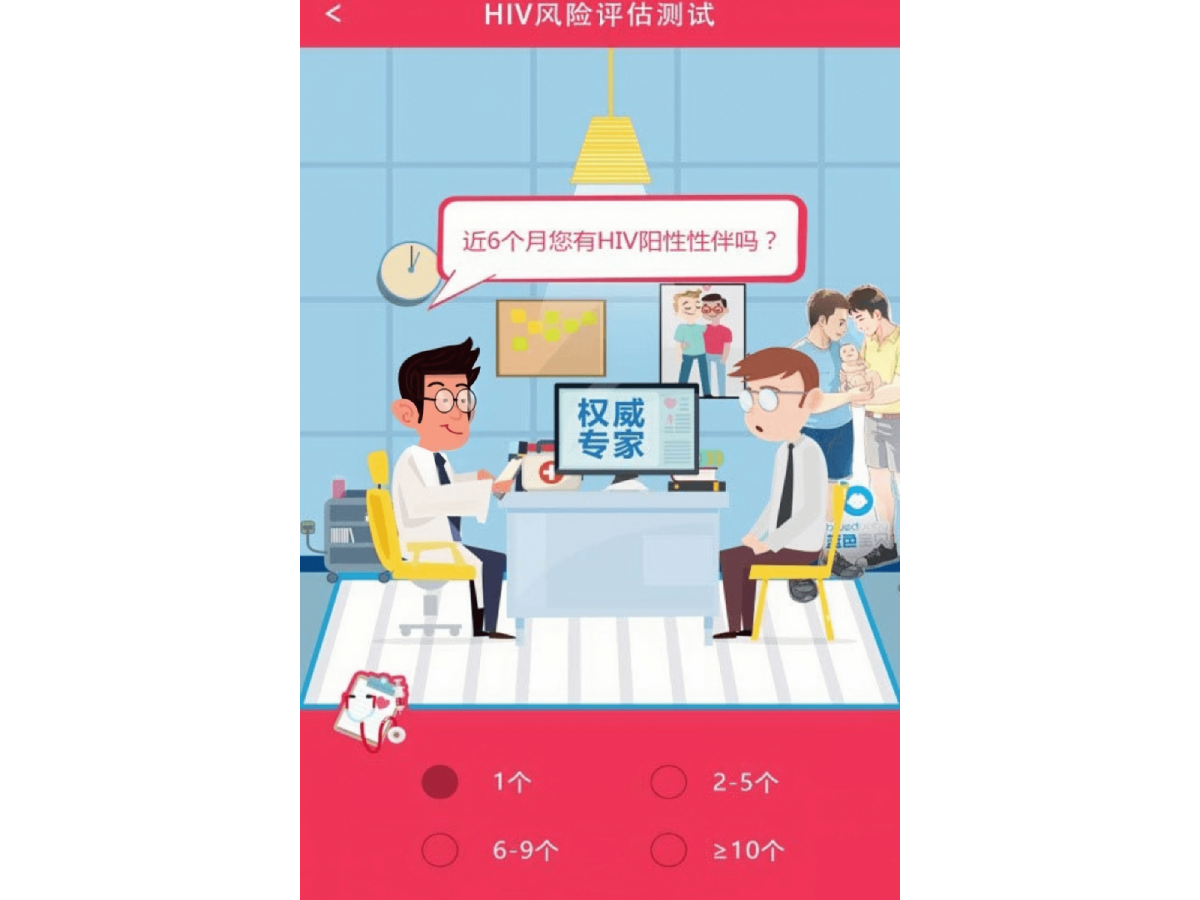
An example of 1 of the 8 items in the HIV RISK Assessment tool. The text reads: “How many HIV-positive homosexual partners did you have in the past 6 months?” Participants would then choose one of the responses: “1,” “2-5,” “6-9,” or “more than or equal to 10”.

Recruitment messages were privately sent through the app to Blued users from a Blued administrative account. The message briefly introduced the study and provided a link to participate. MSM who clicked the link were directed to eligibility screening, the consent form, and the baseline survey. The baseline questionnaire covered demographics, HIV testing history, GSN app use, and sexual risk behaviors. Group 1 and group 2 took the HIV RISK Assessment tool at baseline and 6 months after randomization. The HIV RISK Assessment tool was based on a validated, 8-item questionnaire developed in China [10,27]. Briefly, the tool incorporates the following variables (Figure 1): number of homosexual partners (anal or oral intercourse), number of HIV-positive homosexual partners, frequency of UAI with a man, frequency of commercial male sexual behaviors, diagnosis of sexually transmitted diseases, sex position during anal sex with a man, frequency of recreational drug use, and frequency of group sex with men. All variables were reported for the previous 6 months.

**Figure 2:**
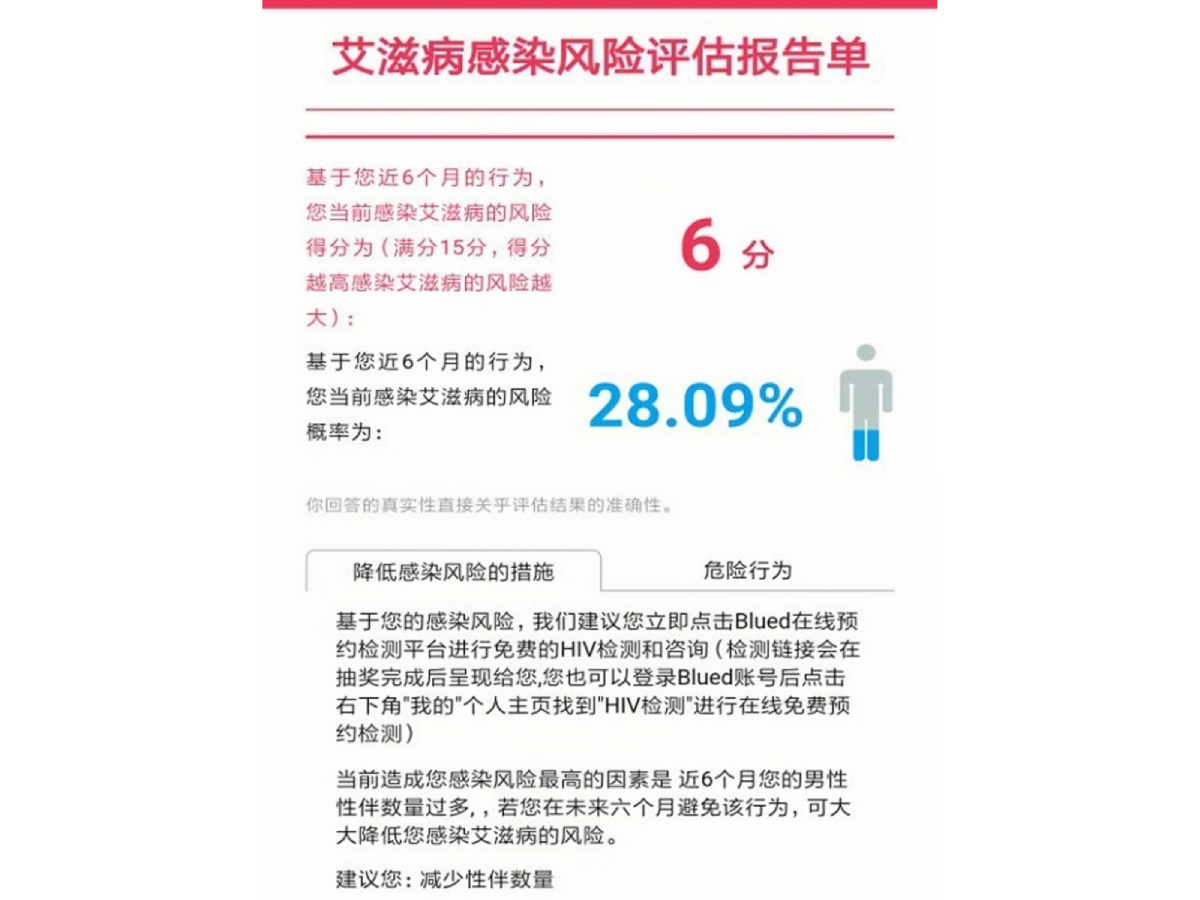
Tailored prevention messaging based on the participant’s risk assessment score. The text reads: “HIV risk assessment report: your current HIV infection risk score is 6 (the total score is 15), and your probability of HIV infection is 28.09% according to your answers to the HIV risk assessment tool. Measures you should take include ordering an offline HIV test in one of the app-based clinics by clicking the button and reducing the number of homosexual partners.”.

Group 1 also received tailored feedback based on their responses to the HIV RISK Assessment tool, which included their HIV risk score, probability of acquiring HIV, high-risk sexual behaviors contributing to their risk of HIV infection, and personalized measures to reduce their risk of HIV infection. An example of this bespoke feedback is captured in Figure 2. Participants in the control group did not receive HIV risk assessment or feedback through the validated tool and instead answered 8 questions on their HIV knowledge based on a questionnaire established by the Chinese CDC at baseline and 6 months after randomization [28].

All participants, regardless of randomization, received a link at baseline and 6 months post randomization to sign up for free HIV testing at convenient drop-in testing sites managed by Blued. If participants scheduled an HIV test, they would receive an SMS text message confirming the location and time of the appointment. Only rapid screening tests were conducted at Blued drop-in testing sites, and those who tested positive were referred to the appropriate district CDC in Beijing for further HIV confirmatory testing by Western blot test and were engaged in antiretroviral therapy, if appropriate. The number of rapid HIV tests taken by participants was collected by working staff at drop-in testing sites run by Blued 1 year after randomization.

Follow-up was conducted at 1 month, 3 months, 6 months, and 12 months following randomization, and the same baseline questionnaire was to be completed, excluding demographic information. The baseline survey was not administered at 12 months post randomization due to low follow-up rates. All questionnaires were conducted on the internet through a unique website developed for the study. If participants did not respond to the initial follow-up surveys, a second link was sent as a reminder 1 week later. Participants were considered lost to follow-up if they had not responded to the second reminder within 1 week.

### Outcomes

The primary outcome of interest was the cumulative number of rapid HIV tests taken over 12 months, described as the mean number of HIV tests per person who attended Blued testing sites for testing per year. The secondary outcome was self-reported UAI at each follow-up, which was assessed by asking “In the past three months, how often did you use condoms for anal sex with a man?” with 4 options: never, sometimes, often, and always. The first 3 options were classified as UAI, and the last as non-UAI. The proportion of UAI was calculated as the number of participants who reported UAI in the past 3 months divided by the number of participants who completed at least one follow-up.

### Statistical Analysis

The primary outcome of this study was the mean number of rapid HIV tests per person per year. We estimated a sample size of 1500 participants (500 per group) was needed to detect a difference of 10% or more in the intervention groups, achieving 90% power at a 2-sided significance of 0.05. The sample size calculation assumed that those in the control group receive an average of 1 HIV test per year, based on a previous study on GSN app users in China [29]. A 30% loss to follow-up during the study was assumed in the power analysis [30].

The mean number of rapid HIV tests per person per year in each group was calculated and analyzed using zero-inflated Poisson regression analysis, estimating the ratios of HIV testing rates in the 2 intervention groups compared to the control group, and reported as incident rate ratios (IRRs) and 95% CIs. The primary analysis was conducted with the intention-to-treat analysis. The proportion of participants who self-reported UAI at each follow-up (1 month, 3 months, and 6 months after randomization) was compared between the study groups using longitudinal, multilevel logistic regression models to account for repeated measures with each individual. The within-individual variable was time (baseline and follow-ups), and the interindividual variable was the intervention group. Adjusted multilevel logistic regression modeling evaluated differences in UAI among study groups. UAI analyses were done with the modified intention-to-treat analyses, excluding those who were never followed.

Descriptive statistics for demographic characteristics, HIV testing history, and sexual risk behaviors were calculated using baseline data and then compared with chi-square tests for categorical variables between those who finished at least one follow-up and those who did not. All statistical analyses were performed with SAS software (version 9.3; SAS Institute, Inc.), and a *P* value of less than .05 was considered statistically significant.

### Role of the Funding Source

The study funders had no role in the study design, data collection, data analysis, explanation of results, or manuscript drafting. The corresponding authors had full access to all study data and had final responsibility for the decision to submit it for publication.

### Ethics Approval

Ethics approval was obtained from the review board of the National Center for HIV/STD Control and Prevention, China CDC (NCAIDS/China CDC, Ethical Approval Number X170623469). Digitally informed consent was retrieved from all participants upon enrollment in the study. All data collected from participants were anonymous**.** Participants who completed each survey received a digital gift card worth RMB 40 (US $5.55).

## Results

**Figure 3:**
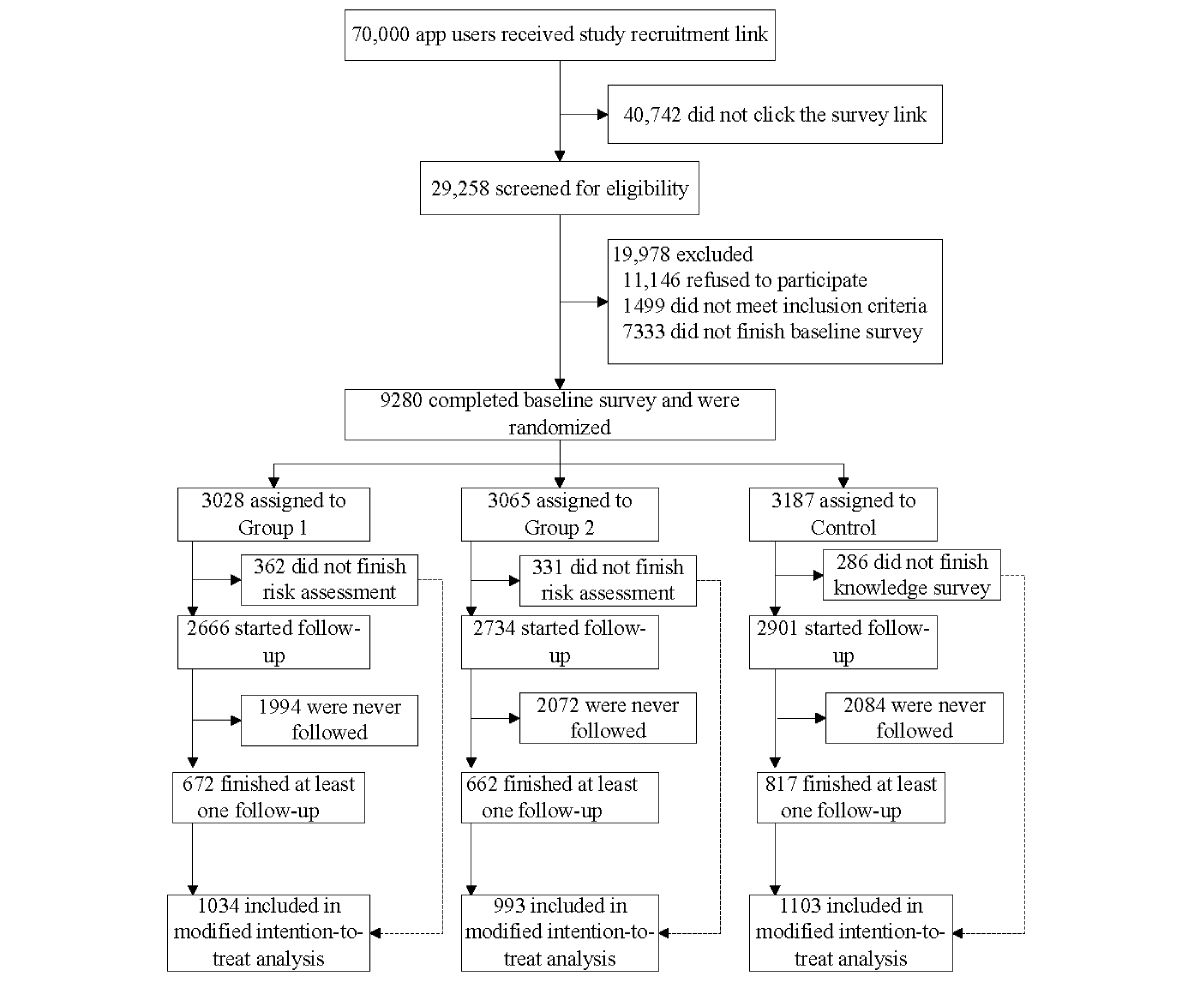
The study flowchart describing the process of recruitment, screening, and the number of participants eligible for analysis.

A total of 70,000 Blued app users received a recruitment link containing information about the study. Of these 70,000 users, 29,258 were screened for eligibility, and 9280 MSM met the inclusion criteria. Following randomization, group 1, group 2, and control groups consisted of 3028, 3065, and 3187 participants, respectively (Figure 3). Of the 3028 participants in group 1, 1034 (34.1%) finished at least one follow-up; of the 3065 participants in group 2, 993 (32.4%) finished at least one follow-up; and of the 3187 participants in the control group, 1103 (34.6%) finished at least one follow-up.

Table 1 summarizes the baseline characteristics of the 3 study groups. More than half (5965/9280, 64.28%) of participants were aged between 18 and 29 years old, almost all (8558/9280, 92.22%) were of Chinese Han ethnicity, 68.09% (6319/9280) completed at least college, 79.34% (7363/9280) were single during the study period, and 63.55% (5897/9280) of participants had their first sexual encounter with a man at ages between 20 and 29 years. Only 35.5% (3294/9280) of participants reported having a convenient HIV testing site nearby their homes; 44.36% (4117/9280) had never performed HIV self-testing; and 55.8% (5178/9280) had never received HIV facility–based HIV testing. Baseline characteristics were balanced across the 3 study groups. Participants who completed at least one follow-up were younger and more likely to have access to HIV testing sites and receive HIV self-tests and facility-based tests within 1 year than those who did not (Multimedia Appendix 1).

As depicted in Table 2, group 1 reported the highest number of HIV tests, with 391 total over the study period, followed by group 2 (n=352) and the controls (n=295). Of those who received HIV tests at follow-up, the mean (SD) number of HIV tests per person was 2.51 (2.18) in group 1, 2.01 (1.94) in group 2, and 1.72 (1.44) in the control group. The mean number of HIV tests in group 1 was significantly higher than the controls (IRR 1.32, 95% CI 1.09-4.58; *P*=.01), but this was not statistically significantly higher for group 2 (IRR 1.06, 95% CI 0.86-1.30; *P*=.60).

**Figure 4:**
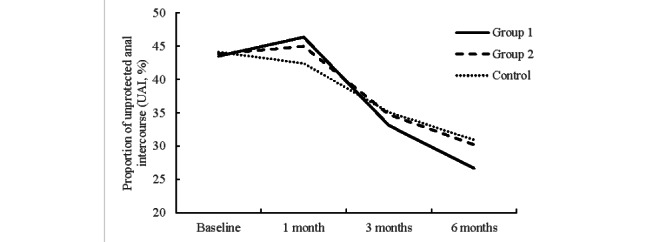
The proportion of self-reported UAI at baseline, 1 month, 3 months, and 6 months after the intervention, by intervention arm.

The proportion of self-reported UAI per study arm is shown in Figure 4. At baseline, the proportion of UAI in the past 3 months was 43.53% (1318/3028) in group 1, 43.86% (1345/3065) in group 2, and 44.14% (1407/3187) in the control arm. At 1-month follow-up, UAI increased to 46.37% (330/713) in group 1 and 44.99% (298/663) in group 2 but declined to 42.42% (316/744) in the control arm. At 3 months, 33.11% (98/295) of group 1, 34.74% (98/283) of group 2, and 35.08% (107/303) of the control reported UAI. At 6 months, the proportion of UAI in group 1 decreased to 26.67% (99/371). Reported UAI also decreased to 30.22% (109/360) in group 2 and 30.95% (117/377) in the control arm.

The proportion of self-reported UAI decreased in all groups over the study period. As seen in Table 3, MSM in group 1 reported a faster rate of decrease in UAI from baseline to the 6-month follow-up (adjusted odds ratio 1.02, 95% CI 0.83-1.10; *P*=.55) compared with the controls, while MSM in group 2 had the same decrease (adjusted odds ratio 1.00, 95% CI 0.82-1.09; *P*=.98) compared with controls, though neither of these findings were statistically different.

**Table 1:** Baseline characteristics of the study participants by randomization arm.

		Total (n=9280), n (%)	Group 1 (n=3028), n (%)	Group 2 (n=3065), n (%)	Control (n=3187), n (%)
**Age (years)**					
	18-24	2962 (31.92)	958 (31.64)	1009 (32.92)	995 (31.22)
	25-29	3003 (32.36)	959 (31.67)	991 (32.33)	1053 (33.04)
	30-34	1686 (18.17)	560 (18.49)	548 (17.88)	578 (18.14)
	35-39	842 (9.07)	296 (9.78)	255 (8.32)	291 (9.13)
	≥40	787 (8.48)	255 (8.42)	262 (8.55)	270 (8.47)
**Ethnicity**					
	Han	8558 (92.22)	2796 (92.34)	2828 (92.27)	2934 (92.06)
	Other	722 (7.78)	232 (7.66)	237 (7.73)	253 (7.94)
**Highest education**					
	College or higher	6319 (68.09)	2041 (67.4)	2090 (68.19)	2188 (68.65)
	Senior high school	2068 (22.28)	693 (22.89)	677 (22.09)	698 (21.9)
	Junior high school or below	893 (9.62)	294 (9.71)	298 (9.72)	301 (9.44)
**Marriage**					
	Single	7363 (79.34)	2423 (80.02)	2429 (79.25)	2511 (78.79)
	Married	1593 (17.17)	504 (16.64)	544 (17.75)	545 (17.1)
	Divorced or widowed	324 (3.49)	101 (3.34)	92 (3)	131 (4.11)
**Sexual debut with a man (years)**					
	<19	2596 (27.97)	832 (27.48)	861 (28.09)	903 (28.33)
	20-29	5897 (63.55)	1945 (64.23)	1953 (63.72)	1999 (62.72)
	≥30	787 (8.48)	251 (8.29)	251 (8.19)	285 (8.94)
**Availability of HIV test sites**					
	Yes	3294 (35.5)	1058 (34.94)	1134 (37)	1102 (34.58)
	No	2931 (31.58)	949 (31.34)	935 (30.51)	1047 (32.85)
	Unclear	3055 (32.92)	1021 (33.72)	996 (32.5)	1038 (32.57)
**Most recent HIV self-test**					
	Within 1 year	4285 (46.17)	1404 (46.37)	1410 (46)	1471 (46.16)
	>1 year	878 (9.46)	288 (9.51)	294 (9.59)	296 (9.29)
	Never	4117 (44.36)	1336 (44.12)	1361 (44.40)	1420 (44.56)
**Most recent facility-based test**					
	Within 1 year	3014 (32.48)	1003 (33.12)	984 (32.10)	1027 (32.22)
	>1 year	1088 (11.72)	360 (11.89)	360 (11.75)	368 (11.55)
	Never	5178 (55.8)	1665 (54.99)	1721 (56.15)	1792 (56.23)

**Table 2:** Number of rapid HIV tests per person per year in Blued app–based clinics among different study groups. The zero-inflated Poisson model was adjusted for age, marriage status, logging onto Blued frequency per week, homosexual debut, available HIV testing locations, and self-perceived HIV epidemic seriousness among men who have sex with men in Beijing.

	Tests, n	HIV tests per person, mean (SD)	IRR^a^ (95% CI)^b^	*P* value	OR^c^ (95% CI)^d^	*P* value
Group 1	391	2.51 (2.18)	1.32 (1.09-4.58)	.01	1.08 (0.85-1.37)	.55
Group 2	352	2.01 (1.94)	1.06 (0.86-1.30)	.60	1.03 (0.73-1.19)	.56
Control	295	1.72 (1.44)	Reference	Reference	Reference	Reference

^a^IRR: incident rate ratio.

^b^Zero-inflated Poisson regression model (number of HIV tests).

^c^OR: odds ratio.

^d^Zero-inflated Poisson regression model (probability of HIV tests).

**Table 3:** Effect of the intervention on the self-reported proportion of unprotected anal intercourse using a mixed effects logistic regression model.

		cOR^a^ (95% CI)	*P* value	aOR^b,c^ (95% CI)	*P* value
**Group**					
	Group 1	1.09 (0.85-1.38)	.51	1.07 (0.84-1.37)	.57
	Group 2	1.10 (0.86-1.41)	.44	1.08 (0.85-1.39)	.52
Time		0.78 (0.71-0.86)	<.001	0.78 (0.71-0.86)	<.001
**Group × time **					
	Group 1 × time	0.96 (0.83-1.10)	.53	1.02 (0.83-1.10)	.55
	Group 2 × time	0.95 (0.83-1.10)	.49	1.00 (0.82-1.09)	.47

^a^cOR: crude odds ratio.

^b^aOR: adjusted odds ratio.

^c^The model was adjusted for age, marriage, and logging onto the Blued app frequency per week. The control group performed as the reference group.

## Discussion

This study found the delivery of HIV risk assessment tools and feedback by a phone-based GSN app improved the number of HIV tests over 1 year among MSM app users in Beijing, China. UAI did not significantly improve compared with controls; however, all UAI declined from baseline to 6 months. Overall, this study suggests app-based, tailored feedback from HIV risk assessment tools could be an effective, low-cost strategy for increasing HIV testing frequency among MSM in China, although longer follow-up is required to establish a definite benefit.

This study shows HIV assessment along with tailored feedback significantly improved the number of HIV tests administered per person, but HIV assessment without feedback did not significantly improve testing compared with controls. This is consistent with the literature purporting that the addition of feedback to HIV assessment has additional benefits [31,32], while HIV assessment in isolation has failed to substantially increase testing rates [31,33]. From this study, just 40% (3712/9280) of individuals had ever engaged in HIV testing at baseline, but those provided with HIV assessment and feedback had a mean test use of 2.51 at follow-up, in comparison with HIV assessment without feedback, which resulted in a mean testing rate of 2.01 per year. This is a drastic improvement and raises the testing of this cohort to the Chinese CDC recommendation of at least once every 3 to 6 months, but it should be noted that our cohort already had HIV testing rates well above the estimated 25% MSM who adhere to recommendations in China [29,34]. Nonetheless, one’s perceived HIV risk is an important factor for motivating HIV testing [12,13] and this RCT highlights that the addition of feedback enforces HIV testing habits. This is likely because feedback provides health care guidance and education that motivates MSM to pursue future steps for HIV prevention, while a risk assessment alone cannot give solutions or promote safe sex behaviors. While this makes sense, other RCTs have found that web-based risk self-assessment tools and tailored feedback may not improve PrEP usage [14], self-reported HIV testing [32], or sexually transmitted infections [35]. These confounding results could be from cross-cultural differences or differences in HIV risk assessment tools used between studies, but they should nonetheless be contextualized in light of the current findings.

Interestingly, the controls also improved their HIV testing rates to the expectations of the Chinese CDC and behaved in a similar fashion to the HIV assessment–only group. In this study, controls received access to a free web-based appointment-making platform to schedule an HIV test. This choice of control is more than what has been offered in other similar studies (eg, relaxation skills [31]) and may have contributed to an intervention-like effect. In addition, the remote-based delivery of free booking services for HIV testing may have interested MSM more than other means of organizing an HIV test, with this added convenience improving testing rates further [36,37]. The anonymous, free, and web-based nature of the appointment platform in a popular GSN app eliminates many barriers to testing and allows for HIV testing in a supportive, culturally safe environment [38]. This study highlights interventions that incorporate social media and supportive and anonymous testing environments as key aspects of increasing HIV testing for MSM in China.

This study also found that HIV risk assessment and feedback significantly reduced the incidence of UAI compared with controls, although it was observed in all 3 groups that UAI declined from baseline. This is in concordance with other studies, which found that completing a risk assessment alone motivated safe changes in sexual behaviors [31,33]. Interestingly, controls were also observed to decrease their incidence of UAI over time. The GSN app Blued periodically disseminates health educational messages to all app users regarding matters such as recreational drug harms or PrEP education, which, alongside the government-based questionnaire, may have been enough of a stimulus to motivate safe behaviors. Another interesting phenomenon observed was the increase in the proportion of UAI from baseline to 1-month follow-up in those receiving feedback with risk assessment. This could be linked to HIV risk assessment scores for MSM, who considered themselves at higher risk for HIV and were then pleasantly surprised by their score, leading to a rebound effect and higher risk behaviors [39]. Thus, appropriate messaging that cautions MSM with low-risk scores should also be considered. Otherwise, the short time between baseline and the first follow-up may not have been long enough to observe an effect on sexual behavior.

This RCT included a large sample of MSM app users in Beijing, China, and investigated interventions that are low-cost, efficient, and scalable for MSM throughout China. The private and simple nature of the intervention likely makes it desirable to MSM, which may increase its success and impact compared to other face-to-face interventions [40]. With the COVID-19 pandemic and its transmission control measures restricting human interaction, other technology-based interventions to address the challenge of HIV testing services have entered the space of public health. For example, an ongoing trial is investigating artificial intelligence chatbots to provide pretest and posttest counseling support among Chinese MSM for promoting HIV self-test uptake [41]. These measures, along with those of this study, may thrive even in the postpandemic era and should be continually evaluated for their efficacy.

Despite the strengths of this study, some limitations should also be acknowledged. First, the number of participants lost to follow-up was high, but this was expected considering web-based interventions are known to have high dropout rates [42,43]. Knowing this, the study recruited in excess to sufficiently power the analysis, so this should be considered an accurate representation of the intervention effect in this population, however, these findings may not be generalizable to people who do not use gay dating apps. Second, HIV testing rates were calculated from data at Blued drop-in testing sites only, thus participants seeking testing elsewhere were not captured in this study and may be underestimated. Despite this, other Chinese studies have shown MSM are more likely to choose nongovernmental organizations like Blued for testing compared to voluntary counseling and testing clinics [44]. Third, UAI was assessed by just 1 self-reported question and may result in recall and social desirability bias. The use of computer-based blinding and intervention through a well-known GSN app may have improved anonymity and encouraged a more open and trusting environment to reduce the impact of this bias.

### Conclusion

This study found GSN app–based interventions that integrate HIV risk assessment with tailored feedback significantly increased HIV testing among MSM in Beijing, China. Integrating these into popular MSM apps would likely increase screening awareness of one’s HIV risk and should be widely considered by stakeholders and government entities.

## Appendix

Multimedia Appendix 1 Baseline characteristics of the study participants.

Multimedia Appendix 2 CONSORT-eHEALTH Checklist (V1.6.2).

## Abbreviations

CDC: Center for Disease Control and Prevention
CONSORT: Consolidated Standards of Reporting Trials
GSN: geosocial networking
IRR: incident rate ratio
MSM: men who have sex with men
PrEP: pre-exposure prophylaxis
RCT: randomized controlled trial
UAI: unprotected anal intercourse
UNAIDS: Joint United Nations Program on HIV/AIDS

## Data Availability

The data sets generated and analyzed during this study are not publicly available as participants’ consent only included data sharing within the project research team.
