# Supplementary material for: Using HIV Risk Self-Assessment Tools to Increase HIV Testing in Men Who Have Sex With Men in Beijing, China: App-Based Randomized Controlled Trial
Source: J Med Internet Res. 2023 Sep 1;25:e45262. doi: 10.2196/45262 (PMC10504623; doi:10.2196/45262)
Supplement: Multimedia Appendix 1 [file jmir_v25i1e45262_app1.docx]

## Multimedia Appendix 1

Table S1. Baseline characters of the study participants between those who finished at least one follow-up and those who did not

|  | Variables | | No follow-up | Finished at least one follow-up | | χ^2^ | *P* |
| --- | --- | --- | --- | --- | --- | --- | --- |
|  |  |  | (N=6150) | (N=3130) |  |  |  |
| **Age (yrs)** | |  |  |  |  | 33.94 | <0.001 |
|  | 18~24 | | 1994 (32.42) | 968 (30.93) |  |  |  |
|  | 25~29 | | 2058 (33.46) | 945 (30.19) |  |  |  |
|  | 30~39 | | 1115 (18.13) | 571 (18.24) |  |  |  |
|  | ≥40 | | 983 (15.98) | 646 (20.64) |  |  |  |
| **Ethnicity** | |  |  |  |  | 2.56 | 0.110 |
|  | Han | | 5652 (91.90 | 2906 (92.84) |  |  |  |
|  | Other | | 498 (8.10) | 224 (7.16) |  |  |  |
| **Highest education** | | |  |  |  | 2.79 | 0.248 |
|  | College or higher | | 4215 (68.54) | 2104 (67.22) |  |  |  |
|  | Senior high school | | 1364 (22.18) | 704 (22.49) |  |  |  |
|  | Junior high school or below 571 (9.28) | | | 322 (10.29) |  |  |  |
| **Marriage** | |  |  |  |  | 8.11 | 0.017 |
|  | Married | | 1024 (16.65) | 569 (18.18) |  |  |  |
|  | Single | | 4928 (80.13) | 2435 (77.80) |  |  |  |
|  | Divorced/widowed | | 198 (3.22) | 126 (4.03) |  |  |  |
| **Sexual debut with a man (yrs)** | | | |  |  | 3.04 | 0.218 |
|  | < 19 | | 1707 (27.76) | 889 (28.40) |  |  |  |
|  | 20~29 | | 3940 (64.07) | 1957 (62.52) |  |  |  |
|  | >= 30 | | 503 (8.18) | 284 (9.07) |  |  |  |
| **Availability of HIV test sites** | | | |  |  | 37.87 | <0.001 |
|  | Yes | | 2049 (33.32) | 1245 (39.78) |  |  |  |
|  | No | | 2003 (32.57) | 928 (29.65) |  |  |  |
|  | Unclear | | 2098 (34.11) | 957 (30.58) |  |  |  |
| **Most recent HIV self-test** | | | |  |  | 15.49 | <0.001 |
|  | Within one year | | 2758 (44.85) | 1527 (48.79) |  |  |  |
|  | >1 year | | 576 (9.37) | 302 (9.65) |  |  |  |
|  | Never | | 2816 (45.79) | 1301 (41.57) |  |  |  |
| **Most recent facility-based test** | | | |  |  | 72.60 | <0.001 |
|  | Within one year | | 1850 (30.08) | 1164 (37.19) |  |  |  |
|  | >1 year | | 676 (10.99) | 412 (13.16) |  |  |  |
|  | Never | | 3624 (58.93) | 1554 (49.65) |  |  |  |
