# Supplementary material for: Using HIV Risk Self-Assessment Tools to Increase HIV Testing in Men Who Have Sex With Men in Beijing, China: App-Based Randomized Controlled Trial
Source: J Med Internet Res. 2023 Sep 1;25:e45262. doi: 10.2196/45262 (PMC10504623; doi:10.2196/45262)
Supplement: Multimedia Appendix 2 [file jmir_v25i1e45262_app2.pdf]

CONSORT-EHEALTH Checklist V1.6.2 Report	Manuscript Number	45262
**(based on CONSORT-EHEALTH V1.6), available at [http://tinyurl.com/consort-ehealth-v1-6].**		
**Date completed**		
1/3/2023 2:43:46		
**by**		
Qianqian Luo		
Using HIV self-risk assessment tools to increase HIV testing in men who have sex with men in Beijing, China: an app-based randomized, controlled trial		
TITLE		
**1a-i) Identify the mode of delivery in the title**		
Yes. Using HIV self-risk assessment tools to increase HIV testing in men who have sex with men in Beijing, China: an app-based randomized, controlled trial		
**1a-ii) Non-web-based components or important co-interventions in title**		
**1a-iii) Primary condition or target group in the title**		
Yes. Using HIV self-risk assessment tools to increase HIV testing in men who have sex with men in Beijing, China: an app-based randomized, controlled trial		
ABSTRACT		
**1b-i) Key features/functionalities/components of the intervention and comparator in the METHODS section of the ABSTRACT**		
Yes. From October 2017 to September 2018, eligible participants were randomly assigned to either Group 1 (access to HIV risk assessment tool with tailored feedback based on their scores), Group 2 (access to HIV risk assessment only), or controls (received government-recommended HIV education).		
**1b-ii) Level of human involvement in the METHODS section of the ABSTRACT**		
**1b-iii) Open vs. closed, web-based (self-assessment) vs. face-to-face assessments in the METHODS section of the ABSTRACT**		
**1b-iv) RESULTS section in abstract must contain use data**		
**1b-v) CONCLUSIONS/DISCUSSION in abstract for negative trials**		
INTRODUCTION		
**2a-i) Problem and the type of system/solution**		
Yes. Despite the high burden of HIV infections in MSM communities, the HIV testing rate among MSM is low in China, where only half of MSM were reported to ever have tested for HIV.		
**2a-ii) Scientific background, rationale: What is known about the (type of) system**		
Yes. This is thought to be from social and psychological barriers, including a lack of trust in facility-based services, hesitancy to use facilities, and anxiety about knowing one's HIV status [11]. Low perceived risk of HIV transmission is also problematic for HIV testing uptake [12-14], with almost 70% of MSM underestimating their risk of HIV [15]. Underestimation of HIV risk can significantly impact HIV testing, leading to under-utilization of preventative services and riskier sexual behaviors [16]. Therefore, effective interventions to expand HIV testing in MSM populations and prevent these deterrents are needed.		
**Does your paper address CONSORT subitem 2b?**		
Yes. Therefore, a randomized controlled trial was conducted where HIV risk assessment and feedback were delivered by a popular GSN, Blued in Beijing, China. The goal of this study was to determine the effect of this intervention on HIV testing numbers and unprotected anal intercourse (UAI) among Chinese MSM.		
METHODS		
**3a) CONSORT: Description of trial design (such as parallel, factorial) including allocation ratio**		
Yes. This double-blinded, triple-arm randomized controlled trial was conducted among MSM in Beijing, China between October 2017 and September 2018.		
**3b) CONSORT: Important changes to methods after trial commencement (such as eligibility criteria), with reasons**		

Not applicable. No change has been made after trial comencement.		
**3b-i) Bug fixes, Downtimes, Content Changes**		
**4a) CONSORT: Eligibility criteria for participants**		
Yes. The inclusion criteria were as follows: MSM who were18 years or older; had anal intercourse with a man in the past six months; self-reported negative or unknown HIV status; resided in Beijing; logged onto the app at least once in the past week before enrollment; and agreed not to share the study materials with others in the study. Participants were excluded if they self-reported injection drug use within six months before enrollment, participated in another online intervention during the study period, or planned to move away from Beijing within the following year.		
**4a-i) Computer / Internet literacy**		
**4a-ii) Open vs. closed, web-based vs. face-to-face assessments:**		
Yes. Recruitment messages were privately sent via the app to Blued users from a Blued administrative account.		
**4a-iii) Information giving during recruitment**		
**4b) CONSORT: Settings and locations where the data were collected**		
Yes. All questionnaires were conducted online via a unique website developed for the study. If participants did not respond to the initial follow-up surveys, a second link was sent as a reminder one week later. Participants were considered lost to follow-up if they had not responded to the second reminder within one week.		
**4b-i) Report if outcomes were (self-)assessed through online questionnaires**		
Yes. The number of rapid HIV tests taken by participants was collected by working staff at drop-in testing sites run by Blued one year after randomization.		
**4b-ii) Report how institutional affiliations are displayed**		
**5) CONSORT: Describe the interventions for each group with sufficient details to allow replication, including how and when they were actually administered**		
**5-i) Mention names, credential, affiliations of the developers, sponsors, and owners**		
**5-ii) Describe the history/development process**		
**5-iii) Revisions and updating**		
**5-iv) Quality assurance methods**		
**5-v) Ensure replicability by publishing the source code, and/or providing screenshots/screen-capture video, and/or providing flowcharts of the algorithms used**		
**5-vi) Digital preservation**		
**5-vii) Access**		
Yes. The message briefly introduced the study and provided a link to participate. MSM who clicked the link were directed to eligibility screening, the consent form, and baseline survey. Group 1 and Group 2 took a HIV RISK Assessment Tool at baseline and six months after randomization.		
**5-viii) Mode of delivery, features/functionalities/components of the intervention and comparator, and the theoretical framework**		
Yes. Group 1 also received tailored feedback based on their responses to the HIV RISK Assessment tool, which included their HIV risk score, probability of acquiring HIV, high-risk sexual behaviors contributing to their risk of HIV infection, and personalized measures to reduce their risk of HIV infection. An example of this bespoke feedback is captured in Figure 2. Participants in the Control group did not receive HIV risk assessment or feedback via the validated tool, and instead answered eight questions on their HIV knowledge based on a questionnaire established by the China CDC at baseline and six months after randomization [26].		
**5-ix) Describe use parameters**		

**5-x) Clarify the level of human involvement**		
**5-xi) Report any prompts/reminders used**		
In our intervention, we did not provide any reminders for the use of the application.		
**5-xii) Describe any co-interventions (incl. training/support)**		
Yes. All participants regardless of randomization received a link at baseline and six months post-randomization to sign up for free HIV testing at convenient, drop-in testing sites managed by Blued.		
**6a) CONSORT: Completely defined pre-specified primary and secondary outcome measures, including how and when they were assessed**		
Yes. The primary outcome of interest was the cumulative number of rapid HIV tests taken over twelve months, described as the mean number of HIV tests per person who attended Blued testing sites for testing, per year. The secondary outcome was self-reported UAI at each follow-up, which was calculated as the number of participants who reported UAI in the past three months divided by the number of participants who completed at least one follow-up.		
**6a-i) Online questionnaires: describe if they were validated for online use and apply CHERRIES items to describe how the questionnaires were designed/deployed**		
**6a-ii) Describe whether and how "use" (including intensity of use/dosage) was defined/measured/monitored**		
**6a-iii) Describe whether, how, and when qualitative feedback from participants was obtained**		
**6b) CONSORT: Any changes to trial outcomes after the trial commenced, with reasons**		
Yes. All questionnaires were conducted online via a unique website developed for the study. If participants did not respond to the initial follow-up surveys, a second link was sent as a reminder one week later. Participants were considered lost to follow-up if they had not responded to the second reminder within one week.		
**7a) CONSORT: How sample size was determined**		
**7a-i) Describe whether and how expected attrition was taken into account when calculating the sample size**		
**7b) CONSORT: When applicable, explanation of any interim analyses and stopping guidelines**		
Yes. The primary outcome of interest was the cumulative number of rapid HIV tests taken over twelve months, described as the mean number of HIV tests per person who attended Blued testing sites for testing, per year. The secondary outcome was self-reported UAI at each follow-up, which was calculated as the number of participants who reported UAI in the past three months divided by the number of participants who completed at least one follow-up.		
**8a) CONSORT: Method used to generate the random allocation sequence**		
Yes. Participants were simply randomized in a 1:1:1 ratio by a computerized randomization algorithm with SAS 9.3 (SAS Institute, Inc. USA) into three groups (Gropu1, Group2, and the Control group). Assignment of group allocations was masked to both the study staff and participants to ensure double-blinding.		
**8b) CONSORT: Type of randomisation; details of any restriction (such as blocking and block size)**		
Yes. Participants were simply randomized in a 1:1:1 ratio by a computerized randomization algorithm with SAS 9.3 (SAS Institute, Inc. USA) into three groups (Gropu1, Group2, and the Control group). Assignment of group allocations was masked to both the study staff and participants to ensure double-blinding.		
**9) CONSORT: Mechanism used to implement the random allocation sequence (such as sequentially numbered containers), describing any steps taken to conceal the sequence until interventions were assigned**		
Yes. Participants were simply randomized in a 1:1:1 ratio by a computerized randomization algorithm with SAS 9.3 (SAS Institute, Inc. USA) into three groups (Gropu1, Group2, and the Control group). Assignment of group allocations was masked to both the study staff and participants to ensure double-blinding.		
**10) CONSORT: Who generated the random allocation sequence, who enrolled participants, and who assigned participants to interventions**		
Yes. Participants were simply randomized in a 1:1:1 ratio by a computerized randomization algorithm with SAS 9.3 (SAS Institute, Inc. USA) into three groups (Gropu1, Group2, and the Control group). Assignment of group allocations was masked to both the study staff and participants to ensure double-blinding.		
**11a) CONSORT: Blinding - If done, who was blinded after assignment to interventions (for example, participants, care providers, those assessing outcomes) and how**		
**11a-i) Specify who was blinded, and who wasn't**		

Yes. Participants were simply randomized in a 1:1:1 ratio by a computerized randomization algorithm with SAS 9.3 (SAS Institute, Inc. USA) into three groups (Gropu1, Group2, and the Control group). Assignment of group allocations was masked to both the study staff and participants to ensure double-blinding.		
**11a-ii) Discuss e.g., whether participants knew which intervention was the "intervention of interest" and which one was the "comparator"**		
**11b) CONSORT: If relevant, description of the similarity of interventions**		
Not applicable. This is an ehealth trial.		
**12a) CONSORT: Statistical methods used to compare groups for primary and secondary outcomes**		
Yes. The mean number of rapid HIV tests per person, per year in each group was calculated and analyzed using zero-inflated Poisson regression analysis, estimating the ratios of HIV testing rates in the two intervention groups compared to the control group, and reported as incident rate ratios (IRR) and 95% confidence intervals (CIs). The primary analysis was conducted with an intention to treat analysis.		
**12a-i) Imputation techniques to deal with attrition / missing values**		
Yes. Adjusted multilevel logistic regression modeling evaluated for differences in UAI among study groups. UAI analyses were done with the modified intention to treat analyses, excluding those who were never followed.		
**12b) CONSORT: Methods for additional analyses, such as subgroup analyses and adjusted analyses**		
Yes. Adjusted multilevel logistic regression modeling evaluated for differences in UAI among study groups. UAI analyses were done with the modified intention to treat analyses, excluding those who were never followed.		
RESULTS		
**13a) CONSORT: For each group, the numbers of participants who were randomly assigned, received intended treatment, and were analysed for the primary outcome**		
Yes. 70,000 Blued-app users received a recruitment link containing information about the study. Of these 70,000 users, 29,316 were screened for eligibility, and 9,280 MSM met the inclusion criteria. Following randomization, Group 1, Group 2, and Control groups consisted of 3028, 3065, and 3187 participants, respectively (Figure 3). Of the 3,028 participants in Group 1, 1,034 (34.1%) finished at least one follow-up, of the 3,065 participants in Group 2, 993 (32.4%) finished at least one follow-up, and of the 3,187 participants in the Control group, 1,103 (34.6%) finished at least one follow-up.		
**13b) CONSORT: For each group, losses and exclusions after randomisation, together with reasons**		
Yes. Figure 3. Study flow chart.		
**13b-i) Attrition diagram**		
**14a) CONSORT: Dates defining the periods of recruitment and follow-up**		
Yes. This double-blinded, triple-arm randomized controlled trial was conducted among MSM in Beijing, China between October 2017 and September 2018. Participants were recruited in October 2017 via the popular Chinese gay dating app, Blued.		
**14a-i) Indicate if critical "secular events" fell into the study period**		
**14b) CONSORT: Why the trial ended or was stopped (early)**		
Yes. Follow-up was conducted at one month, three months, six months, and twelve months following randomization, and the same baseline questionnaire was to be completed, excluding demographic information. The baseline survey was not administered at twelve-month post-randomization due to low follow-up rates.		
**15) CONSORT: A table showing baseline demographic and clinical characteristics for each group**		
Yes. Table 1 summarizes the baseline characteristics of the three study groups.		
**15-i) Report demographics associated with digital divide issues**		
Not applicable. In our study, we recruited participants who were all Blued users.		
**16a) CONSORT: For each group, number of participants (denominator) included in each analysis and whether the analysis was by original assigned groups**		
**16-i) Report multiple "denominators" and provide definitions**		
Yes. The proportion of self-reported UAI per study arm is shown in Figure 4. At baseline, the proportion of UAI in the past three months was 43.53% in Group 1, 43.86% in Group 2, and 44.14% in the Control arm. At one-month follow-up, UAI increased to 46.37% in Group 1 and 44.99% in Group 2 but declined to 42.42% in the Control arm. At three months, 33.11% of Group 1, 34.74% of Group 2, and 35.08% of the Control reported UAI. At six months, the proportion of UAI in Group 1 decreased to 26.67%. Reported UAI also decreased to 30.22% in Group 2 and 30.95% in the Control arm.		
**16-ii) Primary analysis should be intent-to-treat**		

**17a) CONSORT: For each primary and secondary outcome, results for each group, and the estimated effect size and its precision (such as 95% confidence interval)**		
Yes. As depicted in Table 2, Group 1 reported the highest number of HIV tests, with 391 total over the study period, followed by Group 2 (n=352), and Controls (n=295). Of those who received HIV tests at follow-up, the mean (SD) number of HIV tests per person was 2.51 (2.18) in Group 1, 2.01 (1.94) in Group 2, and 1.72 (1.44) in the Control group. The mean number of HIV tests in Group 1 was significantly higher than Controls (IRR=1.32, 95%CI: 1.09-4.58; P=0.01), but this was not statistically significantly higher for Group 2 (IRR=1.06, 95%CI: 0.86-1.30; P=0.60).		
**17a-i) Presentation of process outcomes such as metrics of use and intensity of use**		
**17b) CONSORT: For binary outcomes, presentation of both absolute and relative effect sizes is recommended**		
Yes. The proportion of self-reported UAI per study arm is shown in Figure 4. At baseline, the proportion of UAI in the past three months was 43.53% in Group 1, 43.86% in Group 2, and 44.14% in the Control arm. At one-month follow-up, UAI increased to 46.37% in Group 1 and 44.99% in Group 2 but declined to 42.42% in the Control arm. At three months, 33.11% of Group 1, 34.74% of Group 2, and 35.08% of the Control reported UAI. At six months, the proportion of UAI in Group 1 decreased to 26.67%. Reported UAI also decreased to 30.22% in Group 2 and 30.95% in the Control arm. The proportion of self-reported UAI decreased in all groups over the study period. As seen in Table 3, MSM in Group 1 reported a faster rate decrease in UAI from baseline to six-months (adjusted OR=1.02, 95% CI: 0.83-1.10; P=0.553) compared with the Controls, while MSM in Group 2 had a similar decrease (adjusted OR=1. 00, 95% CI: 0.82-1.09; P=0.984) compared with Controls, though neither of these findings were statistically different.		
**18) CONSORT: Results of any other analyses performed, including subgroup analyses and adjusted analyses, distinguishing pre-specified from exploratory**		
Yes. Table 3. Effect of the intervention on the self-reported proportion of unprotected anal intercourse using a mixed effects logistic regression model		
**18-i) Subgroup analysis of comparing only users**		
**19) CONSORT: All important harms or unintended effects in each group**		
Not applicable. No harms were reported in the intervention.		
**19-i) Include privacy breaches, technical problems**		
**19-ii) Include qualitative feedback from participants or observations from staff/researchers**		
DISCUSSION		
**20) CONSORT: Trial limitations, addressing sources of potential bias, imprecision, multiplicity of analyses**		
**20-i) Typical limitations in ehealth trials**		
Yes. Despite these strengths, some limitations should also be acknowledged. First, the number of participants lost to follow-up was high, as expected considering web-based interventions are known to have high drop-out rates [39, 40]. Despite this, the sample was sufficient to power the analysis adequately and should be considered an accurate representation of the intervention effect. Second, HIV testing rates were calculated from data at Blued drop-in testing sites only, thus participants seeking testing elsewhere were not captured in this study and may be underestimated. Despite this, other Chinese studies have shown MSM are more likely to choose non-governmental organizations like Blued for testing compared to voluntary counseling and testing clinics [41]. Lastly, UAI was assessed by just one self-reported question and may result in recall and social desirability bias. The use of computer-based blinding and intervention via a well-known GSN app may have improved anonymity and encouraged a more open and trusting environment to reduce the impact of this bias.		
**21) CONSORT: Generalisability (external validity, applicability) of the trial findings**		
**21-i) Generalizability to other populations**		
**21-ii) Discuss if there were elements in the RCT that would be different in a routine application setting**		
**22) CONSORT: Interpretation consistent with results, balancing benefits and harms, and considering other relevant evidence**		
**22-i) Restate study questions and summarize the answers suggested by the data, starting with primary outcomes and process outcomes (use)**		

Yes. This study found the delivery of HIV risk assessment tools and feedback by a phone-based GSN application improved the number of HIV tests over one year among MSM app users in Beijing, China. UAI did not significantly improve compared with controls, however, all UAI declined from baseline to six months. Overall, this study suggests app-based, tailored feedback from HIV risk assessment tools could be an effective, low-cost strategy for increasing HIV testing frequency among MSM in China although longer follow-up is required to establish a definite benefit.		
**22-ii) Highlight unanswered new questions, suggest future research**		
Other information		
**23) CONSORT: Registration number and name of trial registry**		
Yes. ClinicalTrials.gov NCT03320239; https://clinicaltrials.gov/ct2/show/NCT0332023.		
**24) CONSORT: Where the full trial protocol can be accessed, if available**		
Yes. https://clinicaltrials.gov/ct2/show/NCT0332023.		
**25) CONSORT: Sources of funding and other support (such as supply of drugs), role of funders**		
Yes. This work was supported by the National Science and Technology Major Project on Prevention and Treatment of Major Infectious Diseases, Including AIDS and Viral Hepatitis from the National Health Commission (2018ZX10721102) and the National Natural Science Foundation of China (72104033).		
**X26-i) Comment on ethics committee approval**		
**x26-ii) Outline informed consent procedures**		
**X26-iii) Safety and security procedures**		
**X27-i) State the relation of the study team towards the system being evaluated**		
